# Efficacy of IV Lidocaine in Reducing Propofol-Related Adverse Events During GI Endoscopy: A Systematic Review and Meta-Analysis

**DOI:** 10.7759/cureus.100457

**Published:** 2025-12-30

**Authors:** Lin Ba, Li-Jie Wang, Hong-Jie Zhang

**Affiliations:** 1 Anesthesiology, Binzhou People's Hospital, Binzhou, CHN; 2 Nursing, Binzhou People's Hospital, Binzhou, CHN

**Keywords:** adverse events, gastrointestinal endoscopy, lidocaine, meta-analysis, propofol, sedation

## Abstract

This systematic review and meta-analysis evaluated the efficacy of IV lidocaine as an adjunct to propofol sedation in reducing propofol-related adverse events during GI endoscopy. Randomized controlled trials (RCTs) comparing IV lidocaine plus propofol vs. propofol alone in adults undergoing GI endoscopy were systematically identified through PubMed, Web of Science, EMBASE, and the Cochrane Library up to December 2025. The primary outcome was the incidence of propofol-related adverse events. Data were pooled using a fixed-effect model, and risk of bias was assessed with the Cochrane Risk of Bias 2 tool. Three RCTs involving 530 patients were included. Meta-analysis revealed that lidocaine significantly reduced the risk of propofol-related adverse events (risk ratio = 0.523, 95% CI: 0.361-0.76) with low heterogeneity (I² = 0%). Sensitivity analysis confirmed the robustness of the findings, although funnel plot asymmetry suggested potential publication bias. In conclusion, IV lidocaine may reduce propofol-related adverse events during GI endoscopy; however, further high-quality studies are needed to confirm these results.

## Introduction and background

GI endoscopy is a commonly performed diagnostic and therapeutic procedure that often requires deep sedation to ensure patient comfort and procedural success. Propofol is widely used for sedation due to its rapid onset and favorable recovery profile. However, its administration is associated with dose-dependent adverse effects, including hypotension, respiratory depression, and injection pain [1,2]. These events can pose significant clinical risks, particularly in elderly patients or those with substantial comorbidities [3].

To minimize propofol requirements, adjunctive agents such as opioids or benzodiazepines are sometimes co-administered; however, these agents may introduce additional risks, including further respiratory depression or delayed recovery [4]. IV lidocaine, a local anesthetic with systemic analgesic, anti-inflammatory, and anti-hyperalgesic properties, has been investigated as a potential alternative adjunct [5]. Its proposed benefits include reducing perioperative opioid consumption, attenuating surgical stress responses, and promoting hemodynamic stability [6]. Several randomized controlled trials (RCTs) have evaluated the combination of lidocaine with propofol for sedation during GI endoscopy [7-9]. To integrate this growing body of evidence and provide a more accurate assessment of its impact on patient safety, a systematic review and meta-analysis was conducted.

The present study aimed to synthesize current evidence from RCTs to determine whether the addition of IV lidocaine to propofol-based sedation reduces the incidence of propofol-related adverse events during GI endoscopy compared with propofol alone. While previous meta-analyses have examined adjunctive therapies in endoscopic sedation, this review was specifically designed with a strict, protocol-defined focus. To ensure a direct and homogeneous comparison, we included only head-to-head RCTs comparing this intervention against propofol alone. This approach prioritizes internal validity and provides a precise estimate of the effect, addressing a defined gap in the existing synthesized evidence.

## Review

Methods

Inclusion and Exclusion Criteria

The criteria for including and excluding studies in this review were predefined. To be eligible, studies had to meet all of the following conditions: (1) the population consisted of adult patients (>18 years old) scheduled for elective GI endoscopy under sedation, with no contraindications to either lidocaine or propofol; (2) the intervention involved IV lidocaine administered as an adjunct to propofol sedation; (3) the comparison group received propofol sedation alone, which could include a placebo control; (4) the outcomes reported included the incidence of propofol-related adverse events, such as hypotension, respiratory depression, hypoxemia, bradycardia, and injection pain; and (5) the study design was an RCT.

Studies were excluded if they were non-randomized, involved pediatric or emergency patient populations, did not report outcomes relevant to this review, or were published in languages other than English.

Search Strategy

A systematic search was conducted in PubMed, Web of Science, EMBASE, and the Cochrane Library. The search included all records from database inception through December 13, 2025. Key search terms included “lidocaine”, “propofol”, “adverse events”, “gastrointestinal endoscopy”, “colonoscopy”, “gastroscopy”, and “randomized controlled trial”. Terms were combined using Boolean operators (AND, OR) to maximize the retrieval of relevant studies. The full PubMed search strategy is provided as a supplement for transparency and reproducibility. Similar strategies were adapted for other databases using appropriate controlled vocabulary and syntax.

Study Selection and Data Extraction

The process of selecting studies and extracting data was conducted independently by two authors. Initially, both reviewers screened the titles and abstracts of all retrieved citations against the eligibility criteria. Full-text articles of potentially relevant studies were then obtained and assessed in detail. Any disagreements that arose during this process were resolved through discussion between the two reviewers or, when necessary, consultation with a third reviewer to reach a consensus. From the final set of included studies, data were extracted using a standardized form. Extracted information included study characteristics, participant demographics, details of the interventions and comparators, measured outcomes, and key results reported by each study.

Quality Assessment

The methodological quality and risk of bias of each included RCT were evaluated using the Cochrane Risk of Bias 2 (RoB 2) tool [10]. This tool assesses five key domains: the randomization process, deviations from intended interventions, missing outcome data, outcome measurement, and selection of the reported result.

Statistical Analysis

The meta-analysis was conducted using R software (version 4.3.0). The primary measure of treatment effect was the risk ratio (RR) with a 95% CI for the incidence of adverse events. Statistical heterogeneity among studies was assessed using the I² statistic [11]. As the calculated I² value was below 50%, indicating low heterogeneity, a fixed-effect model was used to pool the data. Potential publication bias was explored through visual inspection of a funnel plot, and Egger’s test was planned to assess funnel plot asymmetry if a sufficient number of studies (typically 10 or more) were available [12]. However, because only three studies were included, interpretation of the funnel plot for publication bias was considered unreliable. To evaluate the robustness of the overall findings, sensitivity analysis was performed using the leave-one-out method. Assessment of publication bias using funnel plots or statistical tests was not conducted due to the limited number of included studies, which renders such analyses unreliable. This systematic review and meta-analysis was reported in accordance with the Preferred Reporting Items for Systematic reviews and Meta-Analyses (PRISMA) 2020 statement [13].

Results

Study Selection and Characteristics

**Figure 1:**
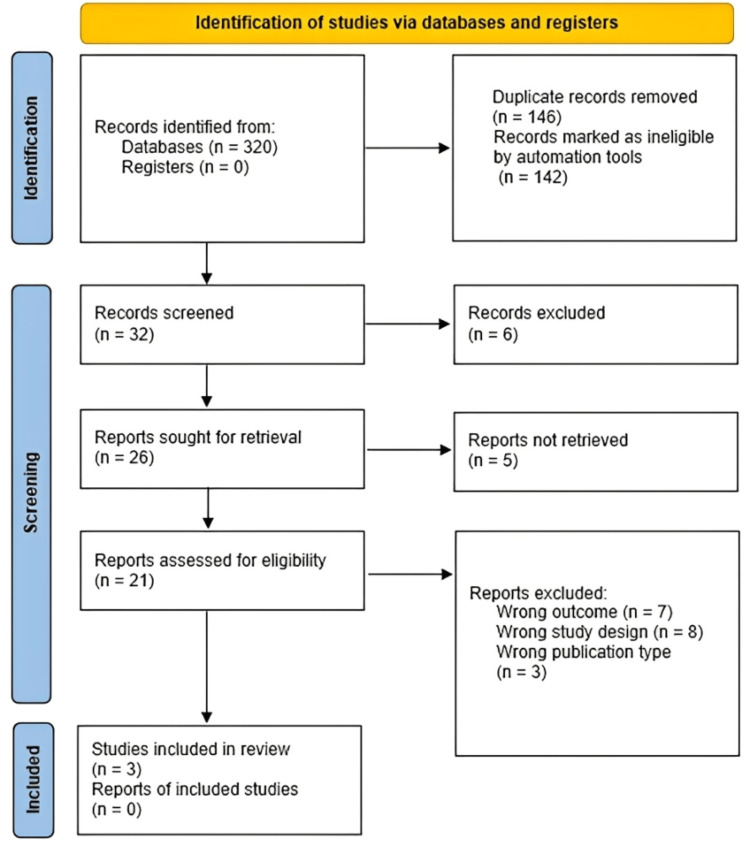
PRISMA inclusion and exclusion flow diagram of the study selection process This figure shows the flow of studies through the systematic review process, including the identification, screening, eligibility, and inclusion stages. PRISMA, Preferred Reporting Items for Systematic reviews and Meta-Analyses

The PRISMA flow diagram illustrates the study selection process (Figure 1). Three RCTs [7-9] met the inclusion criteria for quantitative synthesis, involving a total of 530 patients. Study characteristics are summarized in Table 1. Populations included elderly patients undergoing gastroscopy [9], obese patients undergoing colonoscopy [7], and a general endoscopy cohort [8]. The intervention consisted of IV lidocaine (1.5 mg/kg bolus followed by continuous infusion of 2-4 mg/kg/h) administered with propofol. Control groups received propofol with placebo saline. The primary outcome was a composite of sedation-related adverse events, with definitions provided in the original studies.

**Table 1:** Characteristics of the included studies for the meta-analysis “Group L” refers to the Lidocaine group (intervention), and “Group N” refers to the Normal Saline group (control), as defined in the original study by Hu et al. [9]. ASA, American Society of Anesthesiologists; RCT, randomized controlled trial

Study	Region	Number of participants	Sex	Age	Population	Intervention exposure	Comparator context	Outcome	Study design
Li et al. (2020) [7]	China	90	Male: 52, female: 38	Mean: 44.38 years (SD: 7.13)	Obese patients undergoing a painless colonoscopy	Group L (intervention) received IV lidocaine as an adjunct to propofol sedation, with a loading dose of 1.5 mg/kg and a maintenance infusion of 2 mg/kg/h.	Group N (control) received IV normal saline (0.9%) in the same volume as the lidocaine group, along with propofol for sedation.	Primary: Incidence of sedation-related adverse events. Secondary: Propofol consumption, oxygen desaturation episodes, and satisfaction scores.	RCT
Qi et al. (2025) [8]	China	300	Male: 114 (38%), female: 186 (62%)	Mean: 50.0 years (SD: 9.8)	Patients undergoing GI endoscopy with propofol-based sedation	Intervention group received IV lidocaine as a 1.5 mg/kg bolus followed by a continuous infusion of 4 mg/kg/h throughout the procedure.	Control group received an equivalent volume of normal saline as a placebo during the procedure.	Primary: Incidence of oxygen-desaturation episodes. Secondary: Propofol consumption, body movements, and satisfaction scores.	RCT
Hu et al. (2022) [9]	Shanghai, China	140	Male: 83 (59.3%), female: 57 (40.7%)	Mean: 71.13 years (SD: 4.19)	Elderly patients (≥65 years) undergoing painless gastroscopy with ASA I-II	Group L (intervention) received IV lidocaine via an initial bolus of 1.5 mg/kg followed by a maintenance infusion of 4 mg/kg/h, alongside standard propofol sedation.	Group N (control) received an equal volume of saline intravenously, followed by propofol sedation.	Primary: Composite incidence of sedation-related adverse events. Secondary: Propofol dosing, respiratory events, recovery time, and satisfaction scores.	RCT

Risk of Bias Assessment

**Figure 2:**
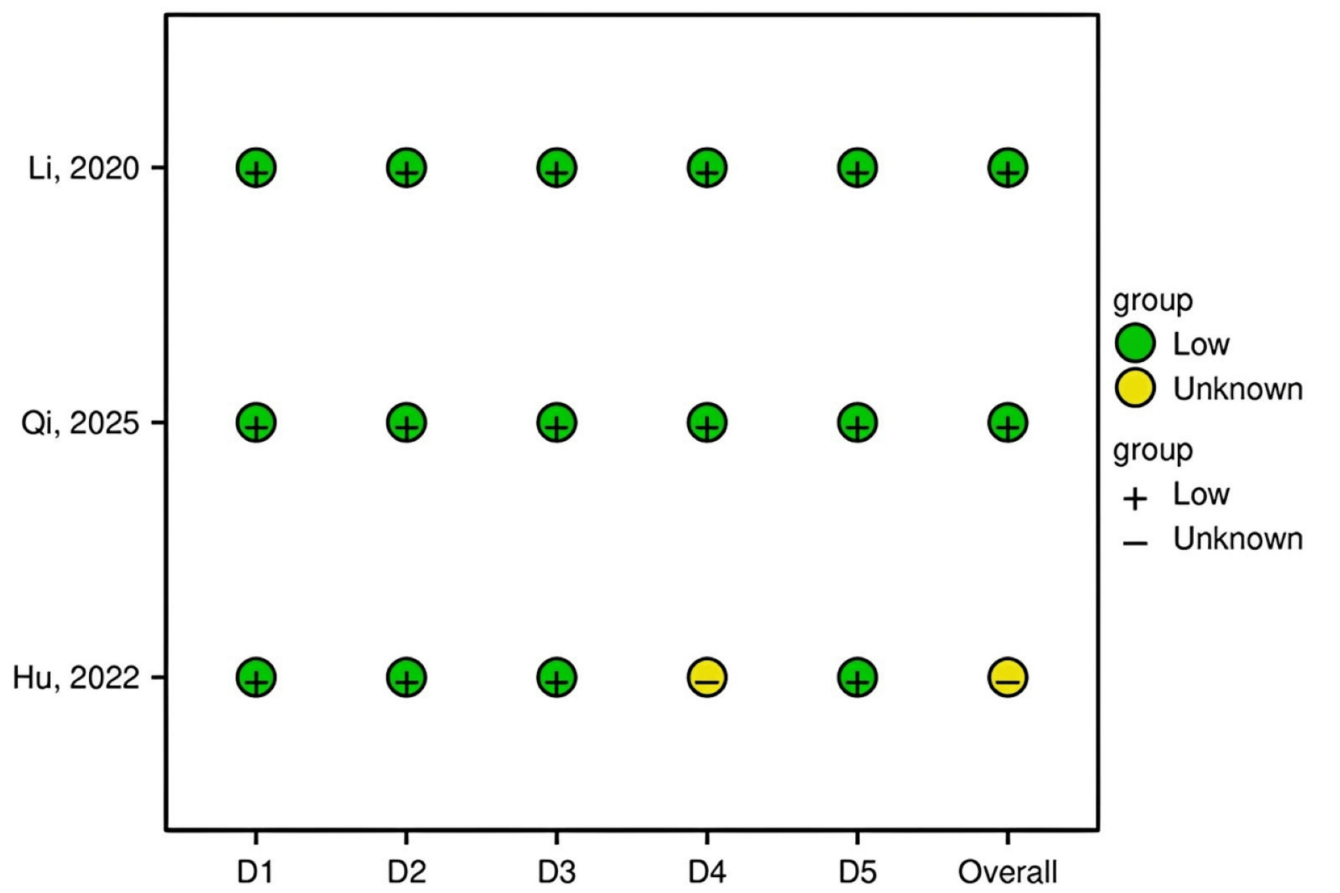
Risk of bias assessment for each included study using the Cochrane RoB 2 tool (traffic light plot) This figure summarizes the methodological quality assessment of studies [7-9] using the Cochrane RoB 2 tool. RoB 2, Risk of Bias 2

**Figure 3:**
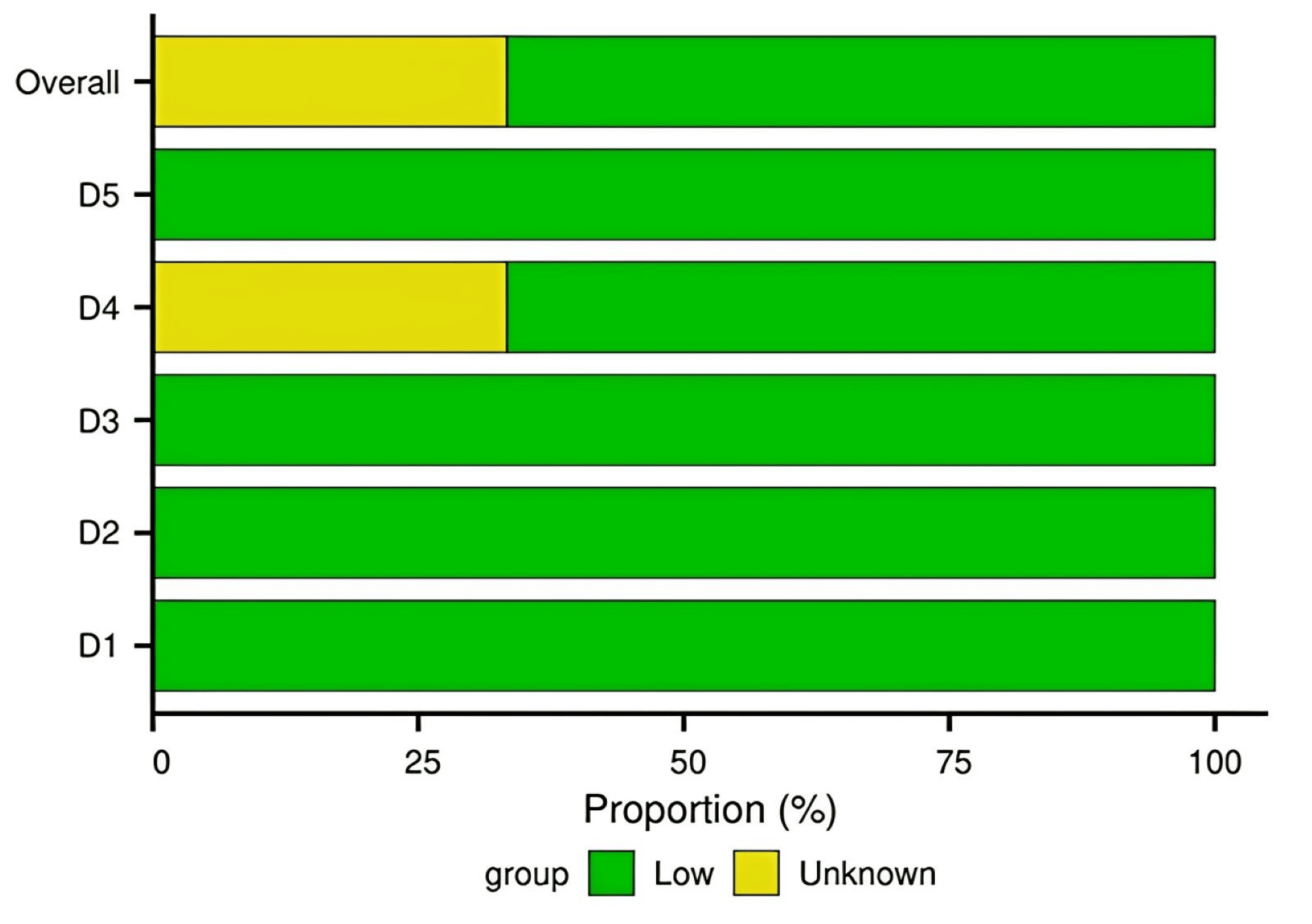
Overall summary of risk of bias across all included studies (weighted bar plot) This traffic light plot depicts domain-level judgments for each individual study: D1, randomization process; D2, deviations from intended interventions; D3, missing outcome data; D4, outcome measurement; D5, selective reporting.

The methodological rigor of the included RCTs was evaluated using the Cochrane RoB 2 tool. Domain-level judgments for each study are presented in a traffic light plot (Figure 2), with an overall summary shown in Figure 3. Two studies [7,8] were judged to have a “Low” risk of bias across all domains. One study [9] raised “Some concerns,” primarily due to insufficient information regarding the blinding of outcome assessors, introducing potential performance bias. Overall, the included studies demonstrated satisfactory methodological quality, supporting the reliability of the pooled data.

**Figure 4:**
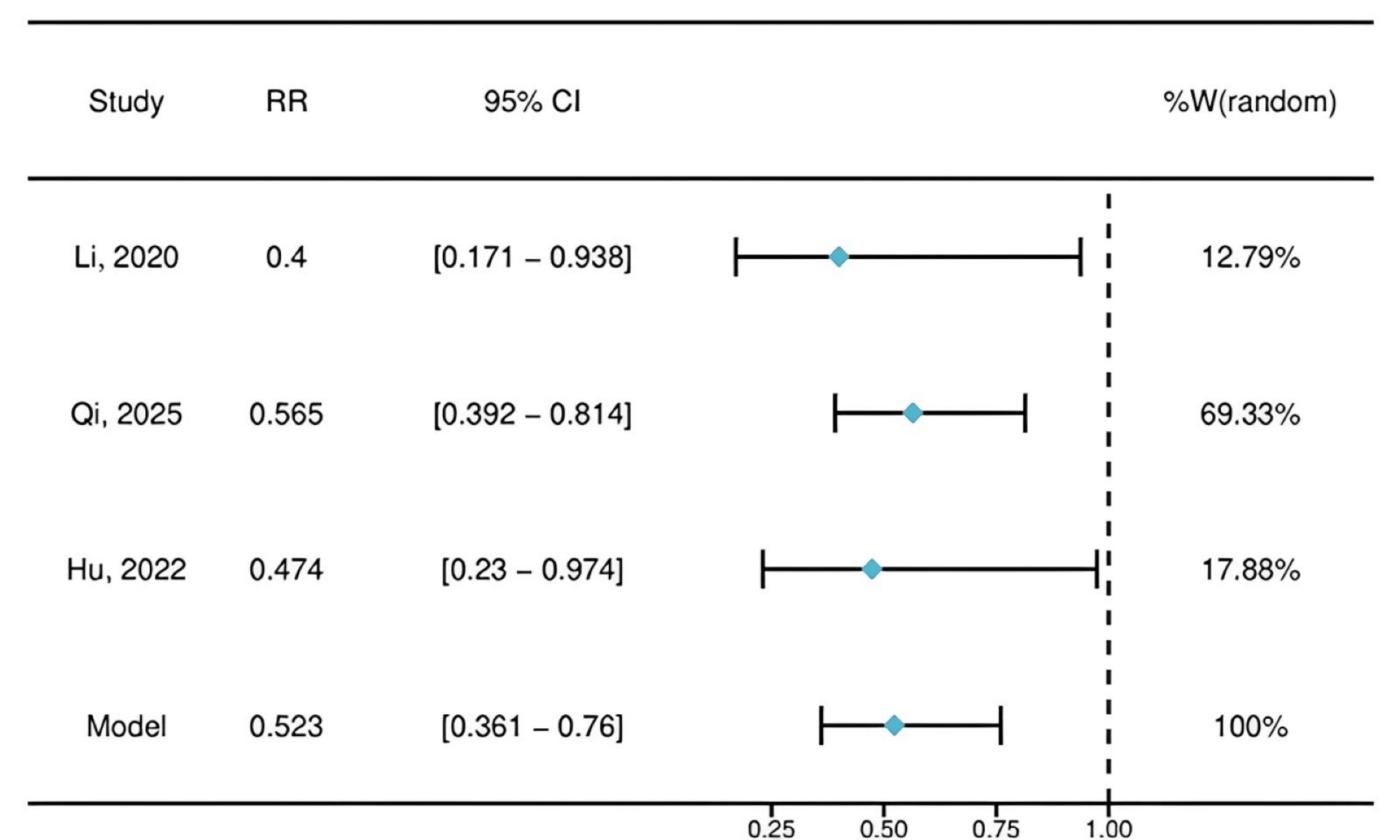
Forest plot of the pooled effect size RRs and 95% CIs for the effect of IV lidocaine on propofol-related adverse events. An RR < 1 favors the lidocaine group, indicating a protective effect. The diamond represents the pooled fixed-effect estimate. RR, risk ratio

The primary meta-analysis comparing the incidence of propofol-related adverse events between the lidocaine and control groups is presented as a forest plot in Figure 4. The pooled RR was 0.523 (95% CI: 0.361-0.76), indicating that prophylactic IV lidocaine was associated with a statistically significant 47.7% reduction in the relative risk of experiencing an adverse event. Notably, all three individual study estimates favored the lidocaine group, and no statistical heterogeneity was observed among the studies (I² = 0%), reinforcing the consistency of the protective effect.

Publication Bias and Sensitivity Analysis

**Figure 5:**
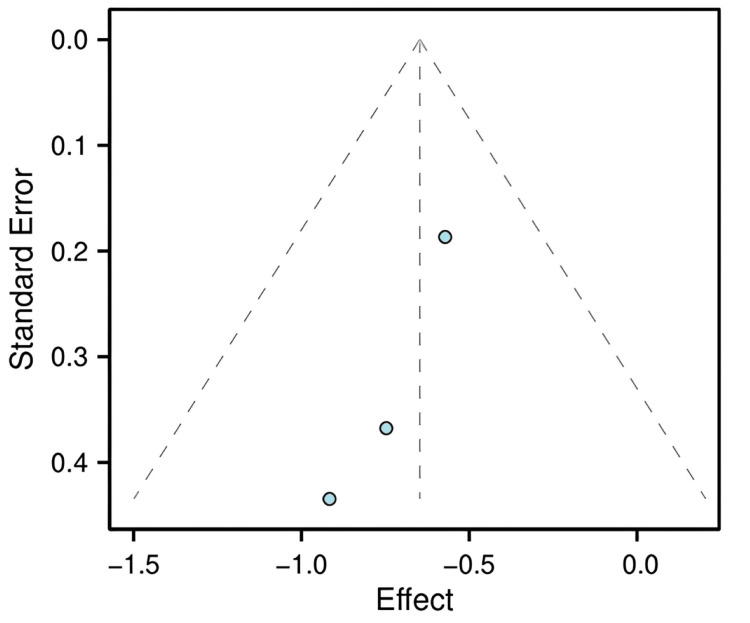
Funnel plot of the studies included in the meta-analysis Visual assessment of potential publication bias. The x-axis represents the effect size (log RR), and the y-axis represents the SE of the effect size. Asymmetry in the plot may suggest publication bias. RR, risk ratio

Although the primary result is robust, the potential for publication bias was assessed. A funnel plot visualizing the SE against the log RR for the three studies is shown in Figure 5. Visual inspection suggests asymmetry, which may indicate that smaller studies with null or negative results are missing from the literature, a common limitation in meta-analyses with a small number of trials. It is important to note that the interpretation of funnel plot asymmetry is highly limited when fewer than 10 studies are included; the observed asymmetry here should therefore be treated with caution.

Due to the limited number of included studies (n = 3), the funnel plot (Figure 5) is presented for completeness but should not be used to formally assess publication bias, as such methods are unreliable with so few studies. To test the robustness of the pooled estimate, a sensitivity analysis was performed using the leave-one-out method (Table 2). This analysis confirmed the robustness of the main finding: the direction of the pooled effect consistently favored the lidocaine group (all pooled RRs < 1) regardless of which study was omitted. Although statistical significance was attenuated (i.e., the 95% CI included 1) when the largest study [8] was excluded, the point estimate remained protective, indicating that the overall conclusion is not critically dependent on any single trial. 

**Table 2:** Sensitivity analysis (leave-one-out method) Results of the leave-one-out sensitivity analysis, assessing the robustness of the pooled effect estimate by omitting one study at a time. TE, pooled treatment effect estimate (RR); lower/upper, lower and upper limits of the 95% CI; tau², estimate of between-study variance; I², proportion of total variability due to heterogeneity RR, risk ratio

Omitted study	Pooled RR	95% CI lower	95% CI upper	Tau²	I²
Li et al. (2020) [7]	0.545	0.221	1.339	0	0
Qi et al. (2025) [8]	0.441	0.153	1.273	0	0
Hu et al. (2022) [9]	0.535	0.109	2.617	0	0

Discussion

This meta-analysis of three RCTs indicates that prophylactic IV lidocaine significantly reduces the risk of propofol-related adverse events by approximately 48% (RR = 0.523, 95% CI: 0.361-0.76) during GI endoscopy. The consistency of this beneficial effect across diverse patient populations, including elderly, obese, and general endoscopy cohorts, strengthens its potential clinical relevance.

The observed reduction in adverse events is supported by plausible biological mechanisms. Lidocaine exhibits systemic analgesic, anti-inflammatory, and membrane-stabilizing properties, which may collectively attenuate procedural stress, reduce propofol requirements [14], and mitigate propofol-induced injection pain and hemodynamic instability [5,15]. By decreasing injection pain and systemic propofol needs, lidocaine may indirectly lessen the reflex hemodynamic and respiratory depression often associated with larger or more rapid propofol boluses. These pharmacological actions align with previous evidence suggesting that lidocaine can enhance perioperative stability and reduce opioid consumption, further supporting its role as a useful adjunct in sedation protocols.

One key strength of this analysis is the low statistical heterogeneity (I² = 0%) among the included studies, supporting the robustness of the pooled estimate. Furthermore, the overall methodological quality of the trials was satisfactory, with two studies rated as having a low risk of bias. Sensitivity analysis confirmed that the direction of the effect remained stable regardless of which study was excluded, reinforcing the reliability of the findings.

Nevertheless, several limitations must be acknowledged. First, this meta-analysis is based on only three RCTs, all conducted in China. While our systematic search included major international databases (PubMed, Web of Science, EMBASE, and Cochrane Library) without language restrictions and involved manual reference screening, no eligible RCTs from other regions met our criteria. The limited number of studies constrains the statistical power and precision of the pooled estimate, and the geographic homogeneity may affect the generalizability of our findings to other populations and healthcare settings. This highlights a critical evidence gap and underscores the need for future multinational trials.

Second, it is important to contextualize the scope of our research question. This review was designed to isolate and evaluate the incremental effect of adding IV lidocaine to a propofol-based sedation regimen compared to propofol alone (or with a placebo). Consequently, variations in propofol dosing regimens between studies, while present, were not the primary focus. More importantly, the consistent use or exclusion of other analgesic adjuncts (e.g., opioids such as fentanyl) was a key element of our inclusion criteria to ensure a direct comparison; studies with unbalanced co-medications between groups were excluded. Therefore, our findings specifically address the benefit of lidocaine within a protocol that minimizes confounding from other systemic analgesics. For clinical translation, future research should investigate optimal propofol-lidocaine synergies and clarify their role within more complex, multi-drug sedation protocols.

Third, although all studies reported a composite outcome of “adverse events,” variations in definitions, monitoring protocols, and component events introduce clinical heterogeneity not fully captured by quantitative measures. Additionally, variability in the lidocaine maintenance infusion rate (2 mg/kg/h vs. 4 mg/kg/h) across studies may introduce further clinical heterogeneity. The absence of a standardized dosing regimen should be considered when interpreting results and designing future trials.

Fourth, our search strategy and inclusion criteria were designed to answer a very specific question, strictly limiting studies to head-to-head RCTs comparing the defined intervention (lidocaine + propofol) vs. control (propofol alone or placebo). This focus enhances internal validity but means our analysis does not encompass the broader literature on alternative adjunctive agents or sedation protocols. Our findings should thus be interpreted as a direct assessment of the incremental benefit of IV lidocaine within this specific context.

Despite these limitations, the clinical implications of our findings are noteworthy. IV lidocaine is a low-cost, widely available, and easily administered intervention that could enhance the safety of propofol-based sedation, particularly in high-risk patients such as the elderly or those with comorbidities. Our results, derived from a synthesis of highly homogeneous evidence, provide a focused and internally consistent answer supporting the judicious use of lidocaine as an adjunct in endoscopic sedation protocols for the specific purpose of reducing propofol-related adverse events.

Future research should address current evidence gaps through larger, multicenter, rigorously designed RCTs employing standardized outcome definitions and monitoring criteria. Studies should also aim to identify optimal dosing regimens, clarify which specific adverse events (e.g., respiratory depression, hypotension, and injection pain) are most effectively prevented, and evaluate the impact of lidocaine on recovery profiles and patient-reported outcomes. Investigations in diverse populations and healthcare systems will further validate and refine these preliminary conclusions.

## Conclusions

This systematic review provides preliminary evidence that IV lidocaine reduces the risk of propofol-related adverse events during GI endoscopy. However, the strength of this evidence is limited by the small number of primary studies and the potential for publication bias. Therefore, while the findings are promising, the routine use of IV lidocaine for this purpose should be considered provisional until confirmed by larger, high-quality trials.
